# Light‐induced complex formation of bacteriophytochrome *Rp*BphP1 and gene repressor *Rp*PpsR2 probed by SAXS

**DOI:** 10.1111/febs.14973

**Published:** 2019-07-12

**Authors:** Miroslav Z. Papiz, Dom Bellini, Kate Evans, J Günter Grossmann, Tony Fordham‐Skelton

**Affiliations:** ^1^ Institute of Integrative Biology University of Liverpool UK; ^2^ STFC Daresbury Laboratory Warrington UK; ^3^ Pharmacy and Biomolecular Sciences Liverpool John Moores University UK

**Keywords:** bacteriophytochrome, complex formation, photo‐induced changes, photosynthesis, SAXS

## Abstract

Bacteriophytochrome proteins (BphPs) are molecular light switches that enable organisms to adapt to changing light conditions through the control of gene expression. Canonical type 1 BphPs have histidine kinase output domains, but type 3 *Rp*BphP1, in the bacterium *Rhodopseudomonas palustris (Rps. palustris)*, has a C terminal PAS9 domain and a two‐helix output sensor (HOS) domain. Type 1 BphPs form head‐to‐head parallel dimers; however, the crystal structure of *Rp*BphP1ΔHOS, which does not contain the HOS domain, revealed pseudo anti‐parallel dimers. HOS domains are homologs of Dhp dimerization domains in type 1 BphPs. We show, by applying the small angle X‐ray scattering (SAXS) technique on full‐length *Rp*BphP1, that HOS domains fulfill a similar role in the formation of parallel dimers. On illumination with far‐red light, *Rp*BphP1 forms a complex with gene repressor *Rp*PpsR2 through light‐induced structural changes in its HOS domains. An *Rp*BphP1:*Rp*PpsR2 complex is formed in the molecular ratio of 2 : 1 such that one *Rp*BphP1 dimer binds one *Rp*PpsR2 monomer. Molecular dimers have been modeled with Pfr and Pr SAXS data, suggesting that, in the Pfr state, stable dimeric four α‐helix bundles are formed between HOS domains, rendering *Rp*BphP1functionally inert. On illumination with light of 760 nm wavelength, four α‐helix bundles formed by HOS dimers are disrupted, rendering helices available for binding with *Rp*PpsR2.

## Graphical abstract

The bacteriophytochrome *Rp*BphP1 forms a complex with repressor *Rp*PpsR2 when stimulated with far‐red light, causing a host of genes responsible for photosynthesis to be switched on. Photo‐isomerization of biliverdin IXα pigments causes long range structural changes from the far‐red (Pfr) to the red (Pr) absorbing state. These changes disrupt a four α‐helix bundle (magenta), making the HOS domain helix‐turn‐helix motif available for complex formation with *Rp*PpsR2.

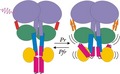

**Abbreviations**
AppA: Blue light photo‐sensor protein
BphP: Bacteriophytochrome
CBD: chromophore‐binding domain
c‐di‐GMP: *bis*‐(3′‐5′)‐cyclic dimeric guanosine monophosphate
DGC: diguanyl cyclase
Dhp: histidine kinase dimerization domain
GAF: GMP‐specific phosphodiesterases‐adenylyl cyclases‐FhlA domain
HK: histidine kinase deomain
HOS: two‐helix output sensor domain
HTH: helix‐turn‐helix motif
PAS: Per‐Arnt‐Sim domain
PCD: photosensory core domain
Pfr: far‐red absorbing state
PHY: photochrome specific domain
Pr: red absorbing state
SAXS: small angle X‐ray scattering
SEC: size exclusion chromatography

## Introduction

**Figure 1:**
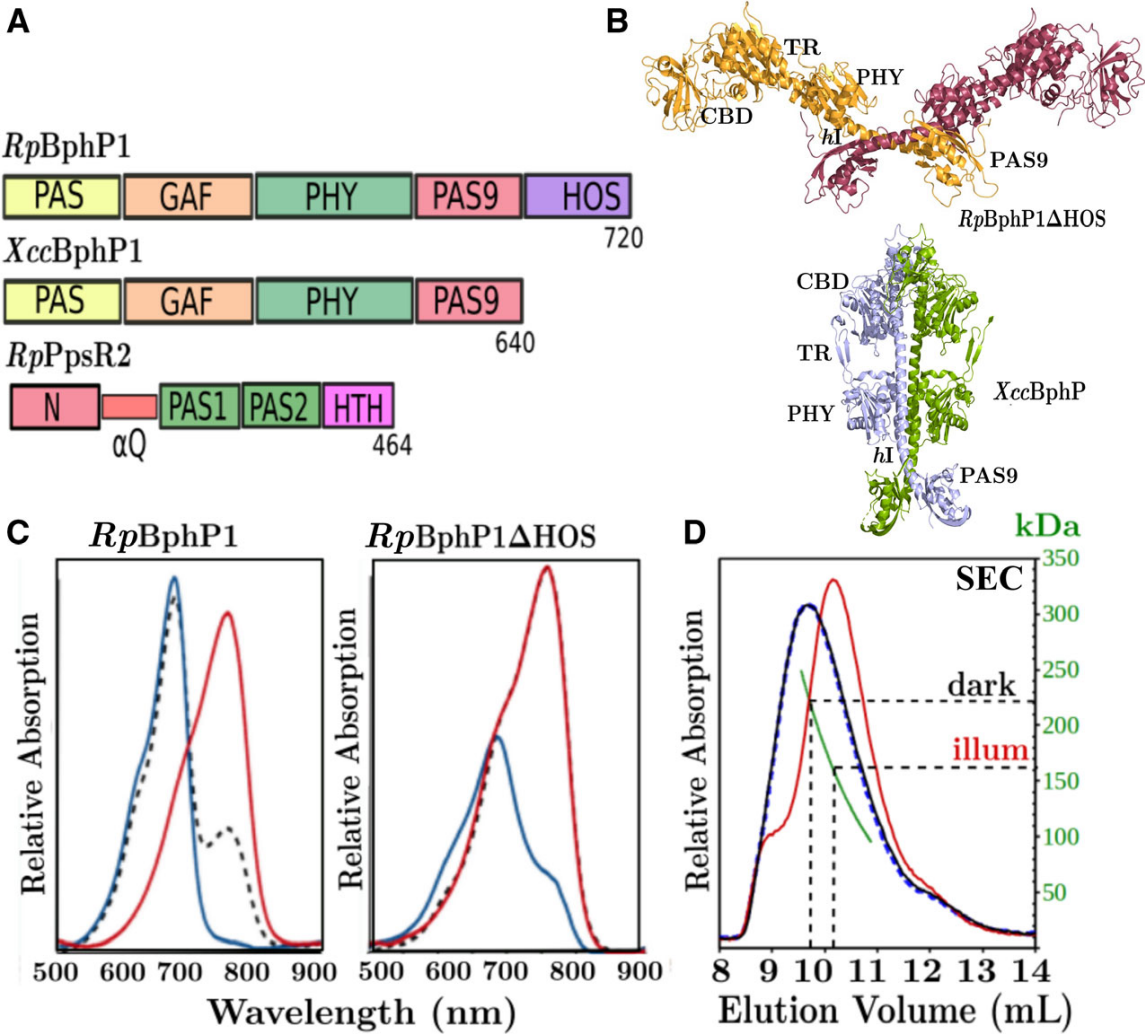
Domain organization, tertiary/quaternary structure, UV‐Vis spectra, and SEC chromatography. Domain organizations of (A) bacteriophytochrome *Rp*BphP1 from *Rhodopseudomonas palustris*, bacteriophytochrome, *Xcc*BphP from *Xanthomonas campestris pv. campestris* and the gene repressor *Rp*PpsR2 from *Rhodopseudomonas palustris*. *Rp*BphP1 and *Xcc*BphP are composed of the chromophore binding domain comprising PAS‐GAF domains (CBD) or the larger photo‐sensory core domain PAS‐GAF‐PHY (PCD), which is the minimal unit for stable photo‐conversion, and C‐terminal output domains PAS9‐HOS and PAS9 respectively. Gene repressor *Rp*PpsR2 is composed of the PAS domain variants N, PAS1, and PAS2. Domains N and PAS1 are connected by a glutamate rich Q‐linker α‐helix (αQ) and the C terminus terminated by the helix‐turn‐helix DNA binding motif HTH. (B) High resolution X‐ray structures of *Rp*BphP1ΔHOS (excluding HOS domain, 4GW9) and *Xcc*BphP (5AKP). Quaternary structures are pseudo anti‐parallel dimers for *Rp*BphP1ΔHOS and parallel dimers for *Xcc*BphP, TR is a tongue region capping the biliverdin chromophore pocket, PHY is a phytochrome specific domain and PAS9 is a type 9 PAS domain. (C) *Rp*BphP1 UV‐Vis spectra of Pfr dark state (red) and the photo‐converted Pr state (blue), a spectrum was also measured 30 min after the start of dark reversion (black dashed line). *Rp*BphP1 dark reversion half‐life is 50 min and for *Rp*BphP1ΔHOS is estimated to be less than 1 min. (D) size exclusion chromatography (SEC) of *Rp*BphP1; apparent dimer molecular weights are ~ 220 kDa for the Pfr dark state (blue) and ~ 160 kDa for the Pr state (red) which was prepared by illumination with 760 nm light. A fully dark reverted Pfr protein (dashed‐blue) is shown to elute as the original Pfr state demonstrating that the process is reversible.

Bacteriophytochrome proteins (BphPs) are photoreceptor proteins that transmit light‐dependent signals to downstream response regulators which affect a variety of light‐dependent physiological functions through the control of gene expression. The photo‐sensing molecule is a tetrapyrrole biliverdin IXα which is covalently attached to a cysteine residue near to the N‐terminus. Biliverdin IXα absorbs light and switches reversibly between the far‐red absorbing Pfr and red absorbing Pr state by isomerization of the D ring pyrrole about the C_14_=C_15_ bond. All BphPs have photosensory core domains (PCDs) at their N termini, comprising PAS‐GAF‐PHY domains, but can have variable C‐terminus output domains. PCD is the minimal structural unit necessary for stable and reversible photo‐conversion. Canonical type 1 BphPs have a histidine kinase output module that consists of a dimerization domain (Dhp) and a kinase domain 1. *Rp*BphP1 from *Rps. palustris* (Fig. 1A) is a type 3 BphP, with a different output domain, that controls the expression of a large cluster of genes involved in photosynthesis 2. The output domain is composed of type 9 PAS domain (PAS9) followed by a two‐helix output sensor (HOS) 3. Although type 1 and type 3 output domains are functionally and structurally different, HOS domains are distant homologs of Dhp, suggesting some structural similarity. *Rp*BphP1, in the red‐absorbing Pr state, modifies the behavior of a cognate response regulator *Rp*PpsR2 3, 4, 5 and we show here that these two molecules form complexes under the influence of far‐red light. Most BphPs form parallel head‐to‐head dimers, as has been shown by cryo‐electron microscopy on full‐length type 1 *Dr*BphP from *Deinococcus radiodurans* 6. High resolution X‐ray structures of the chromophore binding domain (CBD) 7, 8, 9, PCD 10, 11, 12, 13, 14, and PCD‐PAS9 (*Rp*BphP1ΔHOS) 3 have been determined, providing important insights into light‐induced structural changes. The full‐length type 3 *Xcc*BphP has also been determined (Fig. 1A) from *Xanthomonas campestris pv. campestris (Xcc)*; however, this BphP possesses C terminus PAS9 domains only 15. Its biological function is also different, which is to suppress plant infection by attenuation of bacterial virulence systems 16. The X‐ray structures of *Xcc*BphP and *Rp*BphP1ΔHOS have been determined in their Pr and Pfr states, respectively, offering an opportunity to investigate light‐induced changes in type 3 BphPs. A major structural difference in *Xcc*BphP is that the long helix hI, which joins the PHY domain to PAS9, is bowed and the PAS9 domain is rotated outwards from the dimer twofold axis by ~ 30° 3, 15. Another surprising feature of *Xcc*BphP and *Rp*BphP1ΔHOS is their different quaternary organizations (Fig. 1B), head‐to‐head parallel dimer for *Xcc*BphP and a pseudo anti‐parallel dimer in *Rp*BphP1ΔHOS with a cross‐over angle between protomers of ~ 170°. Yet another full‐length BphP structure is type 5 *Is*PadC from *Idiomarina* sp. This BphP allows red‐light regulation of the second messenger *bis*‐(3′‐5′)‐cyclic dimeric guanosine monophosphate (c‐di‐GMP) by diguanylyl cyclase activity in the C terminus GGDEF domain 17, 18. Despite the differences at the C terminus, all BphP types share common light‐induced structural elements that transmit conformation changes to their C‐terminal domains through a long helix (hI).

The target protein of *Rp*BphP1 is the transcription regulator *Rp*PpsR2. This protein is found only in purple bacteria and is responsible for controlling the expression of photosynthetic genes. In purple bacteria, PpsR are loosely conserved, reflecting a high evolutionary divergence of properties 19. Experiments on PpsR, of *Rhodobacter sphaeroides* (*Rba. sphaeroides*) and *Rhodobacter capsulatus (Rba. capsulatus)*, have revealed a common mechanism of binding to a pair of palindromic DNA sequences (TGTN_12_ACA) under oxidizing conditions 20, 21. Formation of a disulfide bond in the C‐terminal HTH domain of PpsR was shown to stimulate binding to DNA 22, 23. However, whereas in *Rba. capsulatus*, PpsR is only redox sensing in *Rba. sphaeroides*, it is also antagonized by a blue light sensing protein AppA 24, 25. In *Rps. palustris* and the closely related *Bradyrhizobium* sp., there are two such proteins, PpsR1 and PpsR2, but in most strains only PpsR1 has a Cys residue, whereas it is missing in PpsR2, indicating different redox behavior 19. An exception to this finding is the *Rps. palustris* strain CGA009, a laboratory strain that has been extensively used to study this bacterial species. CGA009 is not a typical strain because PpsR2 has the residue C428 and thus a similar redox behavior to PpsRs of *Rba. capsulatus* and *Rba. sphaeroides*. In CGA009, *Rp*PpsR2 is under dual control, redox and far‐red light, through interaction with *Rp*BphP1. However, all wild‐type strains studied so far have an R428 within *Rp*PpsR2 and are not redox sensors. They are under the sole control of far‐red light, as will be discussed later 26.

Small angle X‐ray scattering (SAXS) experiments on full‐length *Rp*BphP1 and complex formation with *Rp*PpsR2 will be presented here. Experimental findings indicate that full‐length *Rp*BphP1 forms parallel dimers consistent with canonical BphPs and at odds with the structure of *Rp*BphP1ΔHOS, which we conclude is a pathological dimer caused by HOS domain deletion. Based on the SAXS data, measured in the dark and under illumination with 760 nm light, models of *Rp*BphP1‐Pfr and *Rp*BphP1‐Pr are presented, and are subsequently employed to explain the role played by the HOS domains in the formation of native dimers and *Rp*BphP1:*Rp*PpsR2 complexes.

## Results

### Spectral properties of *Rp*BphP1 and *Rp*BphP1ΔHOS dimers

Samples of *Rp*BphP1 and 70 kDa *Rp*BphP1ΔHOS fragment were prepared. The latter was purified by Ni affinity chromatography of protein, and is a natural degradation product when HOS domains are cleaved after approximately 3‐week period storage Illuminating full‐length *Rp*BphP1 with light of wavelength 760 nm causes complete photo‐conversion to the red Pr state, which dark‐reverts to the Pfr state with a half‐life of ~ 50 min. The *Rp*BphP1ΔHOS fragment exhibits incomplete photo‐conversion with a half‐life much shorter than 60 s (Fig. 1C) and completely dark‐reverts to a Pfr state within 30 min. In comparison, in the same time, the dark‐reverted population of *Rp*BphP1 states is ~ 65:35% Pr/Pfr. Molecular weights determined by size exclusion chromatography (SEC) are ~ 220 and 160 kDa for Pfr and Pr states respectively (Fig. 1D). These states have also been shown to be reversible on dark reversion by SEC. In contrast, no difference is observed between Pfr or Pr states in the SEC profiles of *Rp*BphP1ΔHOS. SEC molecular weight of *Rp*BphP1‐Pfr is anomalously high, whereas the molecular weight of Pr is close to the value calculated from the amino acid composition, implying that the Pr shape is more isotropic compared to Pfr. Greater than expected molecular weights can occur for elongated molecules because they tend to reside in the void volume of the SEC column 27 and this kind of behavior has also been observed for monoclonal antibodies 28. Taken together, SEC data and UV‐Vis spectra suggest that HOS domains play an important role in the creation of stable and reversible Pr states within the chromophore pocket. It is clear that the Pr state, as observed by UV‐Vis spectroscopy, is associated with a reversible change in protein conformation responsible for a relatively large variation in apparent molecular weight, as observed in dark/light SEC data.

### Dark and illuminated SAXS data collection

**Figure 2:**
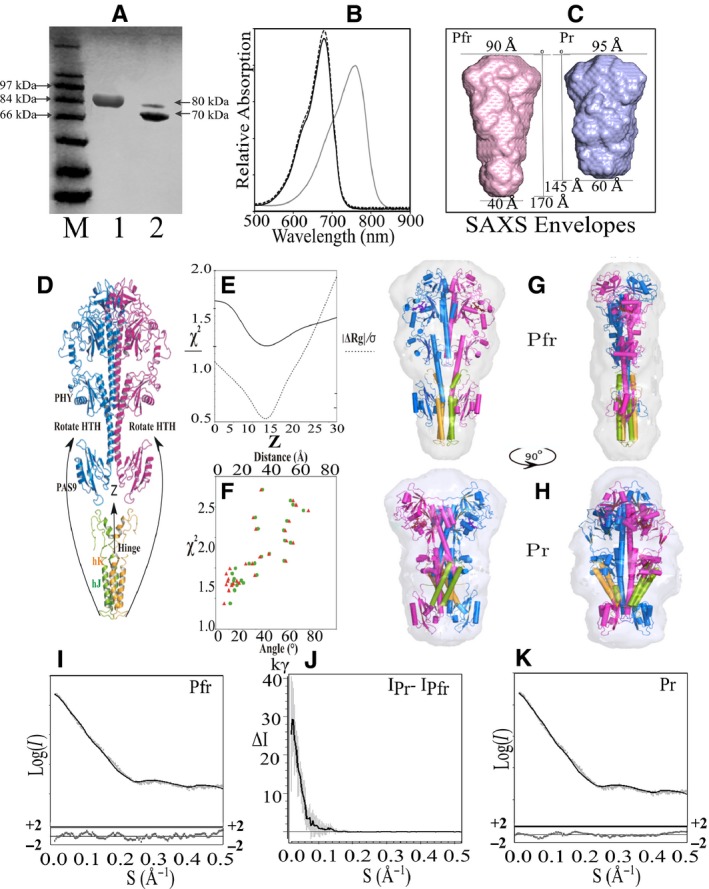
Pfr and Pr SAXS data collection statistics and structure modeling. SDS/PAGE gel (A) of *Rp*BphP1 protein taken from the SAXS sample cell immediately after Pr SAXS data collection, the sample was created by continuous illumination with 760 nm light; M protein markers, lane 1–80 kDa full length protein, lane 2 – by comparison a 3 week old sample showing degradation to a 70 kDa *Rp*BphP1ΔHOS fragment; (B) photo‐conversion UV‐Vis spectrum of *Rp*BphP1 measured offline using the same configuration as on the SAXS beamline; a white light source placed 4 cm from the sample with an intervening interference filter producing 760 nm wavelength light, UV‐Vis optical path‐length was 2 mm and protein concentration 4 mg·mL^−1^. Pfr dark state (gray) and Pr (black) spectrum measured 1 min after illuminating with 760 nm light and 30 min after continuous illumination with 760 nm light (dashed). (C) SAXS *ab*‐initio calculated envelopes, Pfr (pink) and Pr (blue); Pfr has a dimer axis of 170 Å, tapering from 90 to 40 Å; Pr has dimer axis 145 Å, tapering from 95 to 60 Å. (D) A full length Pfr parallel dimer was assembled from *Rp*BphP1ΔHOS (4GW9) monomers and HOS domain dimers were created in Modeller from four sequence aligned Dhp homolog structures, Pr was assembled from a modified *Xcc*BphP and HOS dimers in which HTH motifs were bent at the hinge toward the PHY domain, relative orientations were optimized in CRYSOL. (E) CRYSOL Pfr optimization statistics; χ^2^ (solid) as a function of HOS translation along the common Z twofold axis. The function **|**ΔRg**|/**σ (dashed) was used to test the quality of calculated model Rg with respect to the observed Rg. The function is the modulus difference between observed and calculated Rg divided by its standard deviation σ; (F) Pr structures were tested by rotating HTH motifs into Pr envelope ‘bulge’ region between PAS9 and PHY domains. CRYSOL χ^2^ values are plotted with respect to ‘Angle’ (red triangles) which is an angle defined by the line from the hinge region, through the HTH helices, relative to the fixed line between hinge and helix hF in PHY, ‘Distance’ (green circles) is the corresponding separation in Å between T in HTH and hF. Pfr (G) and Pr (H) models positioned in their respective SAXS envelopes, front and side views. PCD‐PAS9 domains are colored blue/magenta and HOS domains are colored green/orange, the same subunit is colored blue/orange and magenta/green. (I) and (K) are Log(I) versus scattering vector S(Å^−1^) plots for Pfr and Pr states respectively. Solid lines are model calculated SAXS curves superimposed on SAXS experiment scattering points (gray), reduced residuals (*I*(S)_exp _− k.*I*(S)_cal_))/σ(S) are shown which are differences between experimental and calculate intensities divided by experimental standard deviation σ(S), k is a scaling factor; (J) shows intensity differences (ΔI) between illuminated Pr and dark Pfr SAXS data versus scattering vector S. ΔI values are expressed in kilo photons (kγ) units.

**Figure 3:**
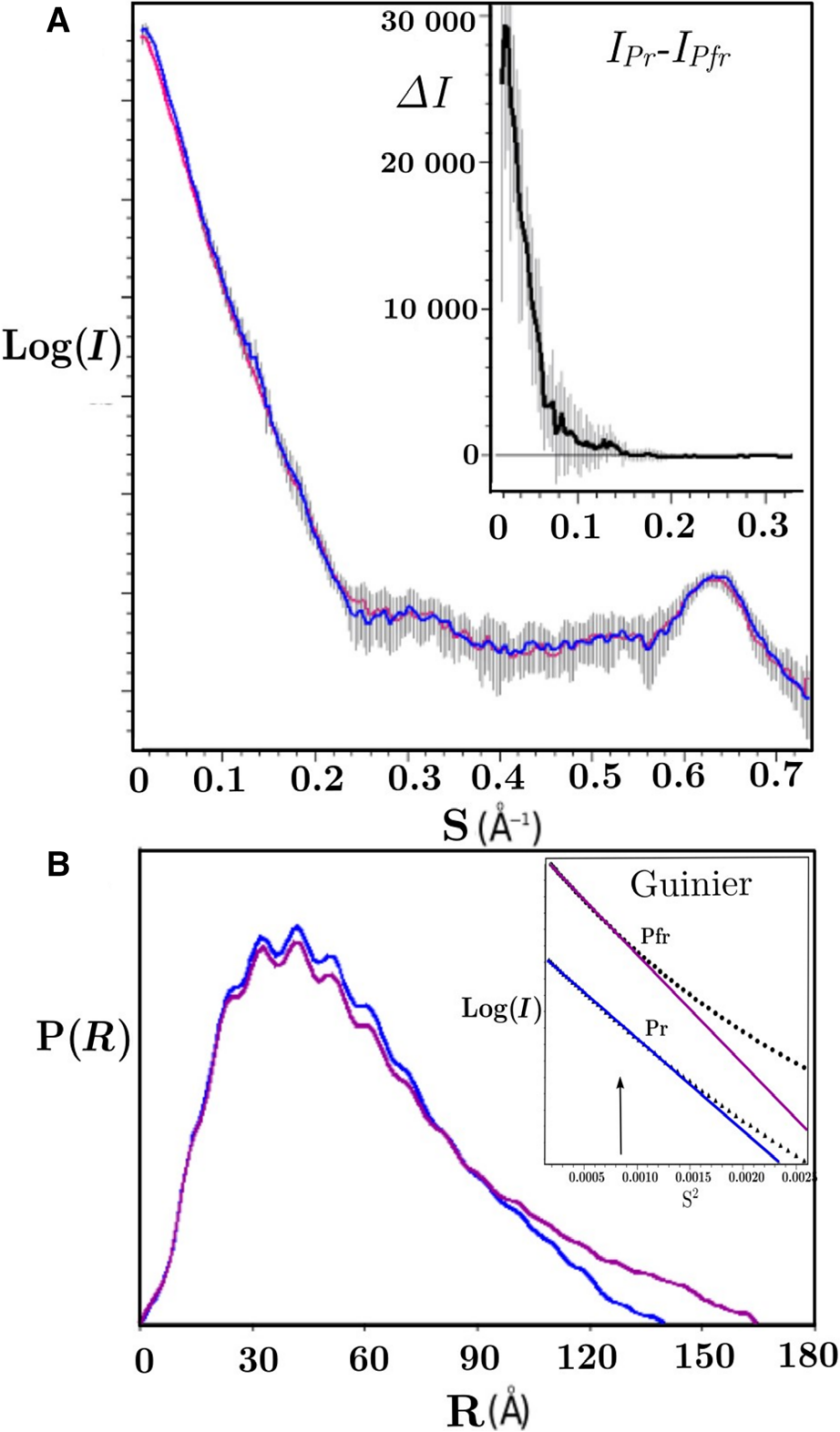
Raw SAXS data log(I(S)), P(R), intensity differences and Guinier plots. Experimental SAXS scattering curves Log(I(S)) versus S(Å^−1^) with error bars (A), Pfr (red) and Pr (blue), inset are intensity differences (*I*_*Pr*_ *‐I*_*Pfr*_) between illuminated and dark SAXS data with superimposed error bars. (B) Pair‐distance distribution function P(R) versus distance R(Å), Pfr Dmax is 160 and 140 Å for Pr. Ripples in P(R) originated from inter helix packing of ~ 10 Å which can also be seen as a scattering curve peak at S = 0.65 Å^−1^. Inset are Guinier plots of Pfr and Pr up to the maximum Guinier resolution S_max_ = 1.3/*Rg* where *Rg* is the radius of gyration 47.8 and 42.8 Å for Pfr and Pr respectively. The Guinier plots are linear down to S = 0.014 Å which corresponds to a real space dimension of 450 Å (~ 3 times Dmax) suggesting the absence of molecular aggregates.

The structure of full‐length *Rp*BphP1, in both Pfr and Pr states, was investigated via the Small Angle X‐ray Scattering (SAXS) method. Given that *Rp*BphP1 holo‐protein degrades to a 70 kDa fragment with the loss of HOS domains (Fig. 2A, lane 2), it was important to obtain a mono‐dispersed sample and thus avoid SAXS data artifacts. A purification protocol was devised to minimize protein degradation by performing every purification step on ice and in darkness. Moreover, both purification and SAXS data collection were completed within 24 h of rupturing bacterial cells. SDS/PAGE of a sample taken directly after Pr SAXS data collection confirmed that the protein is full‐length and mono‐dispersed *Rp*BphP1 (Fig. 2A, lane 1). In addition, gels show only one ~ 80 kDa band and a complete absence of the 70 kDa *Rp*BphP1ΔHOS fragment. To ensure a 100% Pfr, all SAXS measurements were performed in darkness on dark‐purified protein. A 100% Pr state was prepared and maintained in a darkened SAXS experimental beam line by continuous sample illumination with monochromatic light of 760 nm wavelength. Data collection protocols were assessed offline by UV‐Vis spectroscopy to ascertain whether a pure Pr state is attainable using similar conditions to those on the SAXS beam line (i.e., identical optics, light source, sample/light geometry, sample cell thickness, and protein concentration). A sample of 4 mg·ml^−1^ protein concentration placed in a 2 mm UV‐Vis cell path length was shown to undergo photo‐conversion to a 100% Pr state when exposed to λ = 760 nm light within 60 s (Fig. 2B) and this state was maintained by continuous illumination, as evident from the second spectrum (dotted line) measured 30 min later. An *Rp*BphP1ΔHOS UV‐Vis absorption spectrum obtained from an illuminated sample indicates only partial photo‐conversion, with both Pr and Pfr bands observed in the spectrum (Fig. 1C). This property was used to estimate *Rp*BphP1ΔHOS impurity by comparing a SAXS sample to the spectrum comprising of pure *Rp*BphP1 and pure *Rp*BphP1ΔHOS spectra combined in known proportions. Sample impurity could be modeled with the equation A_760_/A_680_ = 0.84Φ + 0.0075 where A_760_/A_680_ is the absorption ratio measured at wavelengths 760 and 680 nm, and Φ is the fraction of *Rp*BphP1ΔHOS impurity. The estimated SAXS sample impurity of Φ = 0.01 ± 0.008 is close to the error limit of measurement; hence, for the purposes of SAXS experiments, the sample can be considered > 99% pure. SAXS data revealed significant differences between states with the radii of gyration (Rg) 47.8/42.8 and Dmax 165 Å/140 Å for Pfr and Pr, respectively (Fig. 3) suggesting that the Pfr molecule is more elongated than the Pr. This is also consistent with the SEC data, which indicates an elongated molecule, as observed by an anomalously high Pfr molecular weight. Molecular masses were determined from Pfr and Pr SAXS data by applying the saxs mow2 program 29, which yielded 163 ± 3.1 kDa for Pfr and 158 ± 2.5 kDa for Pr, indicating that the sample was pure and mono‐dispersed, thus meeting the method requirements. Linear Guinier plots indicate absence of sample aggregation. The Guinier region for *Rp*BphP1 SAXS data is S = 0.014−0.030 Å, corresponding to a lower real spatial limit of 450 Å which is ~ 3 times greater than the longest molecular dimension of 160 Å (Fig. 3). *Ab‐initio* Pfr and Pr envelopes were reconstructed (Fig. 2C) using the data range S = 0.0−0.5 Å^−1^ assuming molecular dimers but not molecular anisometry, as this enabled the automatic *ab‐initio* algorithm to create a two‐fold axis along or across the longest molecular dimension, thus allowing for the possibility of parallel or pseudo anti‐parallel dimers. Calculated envelopes show dimer axes along the longest molecular dimension, which is consistent with parallel dimers, while ruling out pseudo anti‐parallel dimers. Ten Pfr and Pr envelopes were averaged with normalized spatial discrepancies (NSD) 0.65 and 0.82 respectively. The dimensions of Pfr and Pr dimers were 170 and 145 Å, respectively, tapering from 90 to 40 Å, and from 95 to 60 Å (Fig. 2C). In the third dimension, Pfr is 40 Å and Pr is 55 Å, indicating that the Pr dimer is more isotropic in shape than Pfr. Pr and Pfr intensity difference ranged from ~ 30 000 to 1000 photon counts in the scattering range S = 0.0−0.15 Å^−1^ (Fig. 2J). The value S = 0.15 Å^−1^ corresponds to a real spatial dimension of ~ 40 Å and is consistent with the observed Pfr and Pr envelope dimensions, which differ by ~ 30 Å, implying relatively large structural changes.

**Table 1:** SAXS data collection and scattering parameters. Parameters are reported for the dark Pfr and illuminated Pr states.

Data collection parameters	
Instrument	STFC SRS Beam Line 2.1
Beam geometry	Horizontal 5 mm × Vertical 1 mm
Wavelength (Å)	1.54
Camera lengths (m)	1.00 and 4.25
*S* range (Å^‐1^)	0.014–0.730
Exposure time^a^	60 × 1 min
Concentration (mg·mL^−1^)	1 and 4
Temperature (K)	281

	Pfr state	Pr state
Structural parameters		
*I(0)* from *P(r)* (cm^−1^)	0.1113 ± 0.0003	0.1330 ± 0.0004
*Rg* from *P(r)* (Å)	47.84 ± 0.14	42.82 ± 0.11
*I(0)* from Guinier (cm^−1^)	0.1186 ± 0.0005	0.1356 ± 0.0009
*Rg* from Guinier (Å)	47.48 ± 0.28	42.47 ± 0.26
*Dmax* (Å)	165 ± 5	140 ± 5
Porod volume (10^3 ^Å^3^)	144 ± 1.5	136 ± 1.0
Molecular mass calculation		
MoW2 29 from SAXS data (kDa)	163 ± 3.1	158 ± 2.5
Dimer mass from sequence (kDa)	160	
Software employed		
Data reduction	bsl/otoko 50	
Data processing	gnom 51	
*ab‐initio* SAXS envelope analysis	dammin 43	
Validation and averaging	supcomb/damfilt/damaver 44, 52	
Fragment modeling	hhpred/modeller 48, 49	
Computation of model I(S)	crysol 45	
Rigid body refinement	sasref 46	
3D graphics presentation	pymol 53	

a Sixty 2D images were measured on a multi‐wire chamber. Each image was exposed to X‐rays for 60 seconds. The 60 images were checked for radiation damage and averaged into one image. No significant radiation damage was observed so the effective exposure time of the averaged image was 1 h.

### SAXS modeling of *Rp*BphP1‐Pfr structure

**Figure 4:**
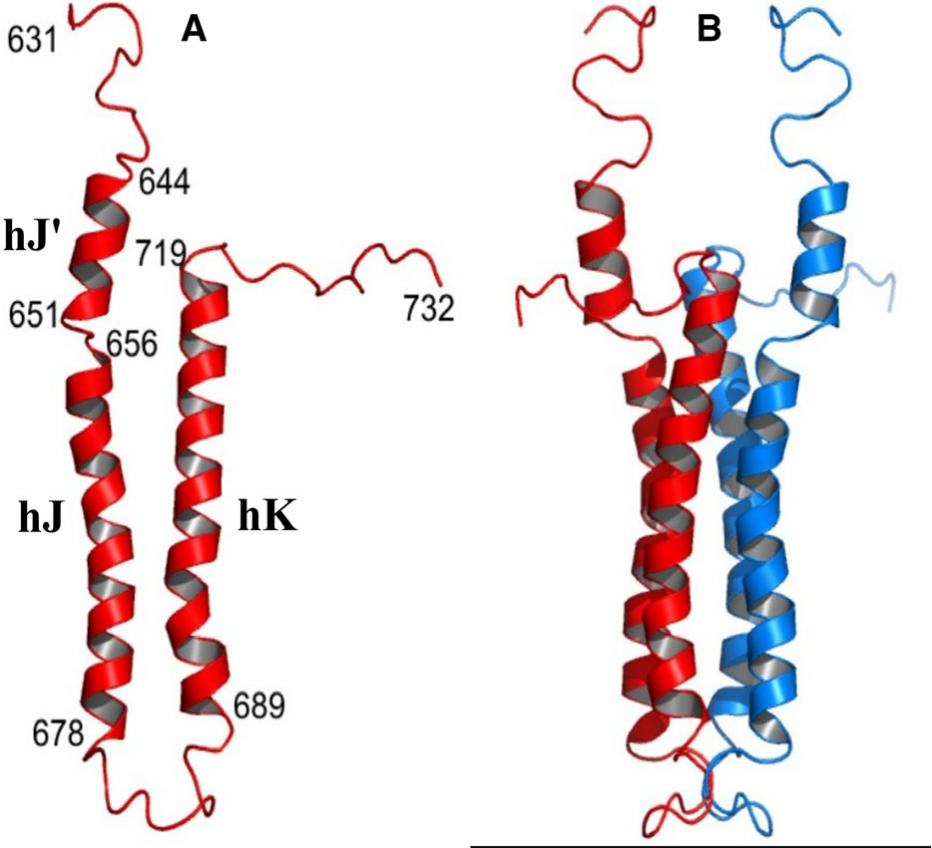
Model of HOS domain. The HOS domain sequence was used to search for homolog PDB structures with *HHpred* toolkit. Four structures (PDBID, 4MT8, 4Q20, 3A0R, 5IDJ) were found, with probabilities ~ 97% and E‐values in the range 7 × 10^‐5^–1.5 × 10^−6^, which are histidine kinase dimerization domains (Dhp) and were used as templates to build HOS domain dimers within the program modeller 49. (A) is a single HOS domain comprising three α‐helices hJ′, hJ, and hK, (B) is a four α‐helix bundle dimer made from two HOS monomers. Random coils were predicted in the sequence regions 632–643 and 720–732. A small α‐helix segment hJ’ was predicted at 644–651 with a break between residues 651–654 followed by a helix‐turn‐helix (HTH) motif which is hJ (656–678), T (679–688), and hK (689–719). In several runs of Modeller, using different Dhp template structures, the amino acid sequence 651–654 was observed to behave as a hinge; the angle between helix axes of hJ′ and hJ varied between 20° and 95°.

*Rp*BphP1 models were developed and optimized to the SAXS data. The core of the model was based on the X‐ray structure of *Rp*BphP1ΔHOS in the Pfr state. Moreover, PCD‐PAS9 domains were built into the parallel dimer structure of *Pa*BphP1‐PCD (3C2W) which is also a bathy‐BphP with 32% sequence identity. To complete a full‐length structure, a HOS domain was built with the program modeller using four sequence‐aligned Dhp homologue X‐ray structures (Fig. 4). The HOS model begins with a random coil (631–643) followed by a short α‐helix (hJ′, 643–651), which is disrupted at residue 651 by the sequence Pro‐Gly, after which it continues into a helix‐turn‐helix (HTH) motif, formed by α‐helices hJ and hK, and ends in a random coil at residues 720–730.

Several Modeller runs with different sets of HOS homologue structures consistently produced a break between hJ′and hJ helices, resulting in a range of angles between helices of 20–95°, indicating that the 651–653 region behaves like a hinge. Full‐length Pfr dimers were created by varying the relative positions of PCD‐PAS9 and HOS domain dimers assuming a common twofold axis. A one‐dimensional translation search of HOS dimers was conducted along the twofold Z axis and was fitted against the SAXS scattering data in the crysol program (Fig. 2D). A χ^2^ fit to the SAXS data is shown in Fig. 2E. A second function, |ΔRg|/σ, was devised to evaluate the consistency between the calculated and experimental radii of gyration. It is defined as the modulus difference between the observed and calculated radius of gyration Rg divided by the experimental standard deviation σ (Fig. 2F). Both functions exhibit well‐defined minima, χ^2^ = 1.4 and |ΔRg|/σ = 0.6, at a Z shift of 13.7 Å relative to an arbitrary starting position. A 3D search was also employed using the rigid body refinement program sasref, which accessed 200 000 models resulting in the best solution with χ^2^ = 1.6, which was within 3.5 Å of the above optimized model. A hydration shell‐optimized CRYSOL calculation provides a fit to the SAXS data of χ^2^ = 0.92 (Fig. 2I) with reduced residuals across the scattering range S (0.0–0.5 Å^−1^) of −1 to +1. An important feature of the optimized Pfr model is that HOS hJ′ helices must slide between hI helices which can form a short four α‐helix bundle, while helices hJ and hK form a longer four α‐helix bundle as observed in homolog Dhp dimerization domains of Histidine Kinases (Fig. 2G). It is not possible to see structural details within the SAXS envelopes, but X‐ray structure models augment this data, indicating how four α‐helix bundles can be best positioned within the envelope. In the case of Pfr helix hJ′ must overlap with hI, to form a short four α‐helix bundle, so that the longer four α‐helix bundle can fully reside within the Pfr envelope.

### Modeling of the *Rp*BphP1‐Pr structure

The *Rp*BphP1‐Pr model was based on the parallel dimer X‐ray structure *Xcc*BphP in the Pr state, modified to the *Rp*BphP1 amino acid sequence and rebuilt in Modeller 15. *Xcc*BphP structure has a shorter hI helix and was extended by three turns. The PAS9 domain in this model is rotated away from the twofold axis by ~ 30°, as it is in *Xcc*BphP, which positions it partially outside the Pr SAXS envelope and reducing PAS9 rotation to ~ 15° positions it completely inside the envelope. Rotation variability is also observed in the structure of *Xcc*BphP, where PAS9 domains deviate from each other by ~ 13° indicating significant flexibility. As the Pr envelope is shorter than the Pfr envelope along the direction of the dimer axis by ~ 25 Å, an extended HOS domain four α‐helix bundle cannot be accommodated within the envelope or reproduce the observed experimental Rg, which results in the |ΔRg|/σ of around 6. A major rearrangement of HTH motifs (hJ and hK bundles) toward the N terminus is required to obtain |ΔRg|/σ < 1. An unaccounted‐for feature in the Pr envelope is a ‘bulge’ situated between PHY and PAS9 domains which is capable of accommodating HTH motifs. A systematic search was performed of HTH orientations over the ‘bulge’ region using rearrangement about the hinge region while leaving hJ’ helices anchored to hI. The behavior of a number of models is illustrated in Fig. 2F, which depicts a plot of CRYSOL χ^2^ values for each model as a function of ‘Angle’ and ‘Distance’. The angular displacement ‘Angle’ is defined by a line through the moving length of HTH relative to a fixed line joining the hinge region to helix hF in the PHY domain. ‘Distance’ is the corresponding spatial displacement of the turn T in HTH to hF. The top five models have χ^2^ =1.4−1.6 with HTH motifs approaching hF with ‘Angle’ in the 6°−12° range and ‘Distance’ in the 11 Å−16 Å range. The best Pr model analyzed in CRYSOL with the optimized hydration shell parameters yields χ^2^ = 0.94. A more extensive rigid body refinement of the HTH position, accessing ~ 200 000 models, provides a solution of χ^2^ = 0.7 which differs from manually refined models by a slightly larger HTH ‘Angle’ displacement of around 20°. Figure 2K indicates that the best model fit to the SAXS data has reduced residuals between ‐1 and +1 across the entire S range. It is clear from exhaustive modeling that the best models have HTH motifs bent toward the PHY domain so that T of HTH approaches helix hF. Such models account for the ‘bulge’ region and the **|**ΔRg|/σ and χ^2^ values are reproduced to values that are less than 1.0. However, accurate positioning of HTH motifs in the ‘bulge’ region is not possible, as reasonable solutions can be obtained over an ‘Angle’ range of 6°–25°. The uncertainty in positioning HTH within the ‘bulge’ is to be expected because these motifs contribute only 14% to the whole structure and thus to the SAXS data.

### *Rp*BphP1‐*Rp*PpsR2 complex

**Figure 5:**
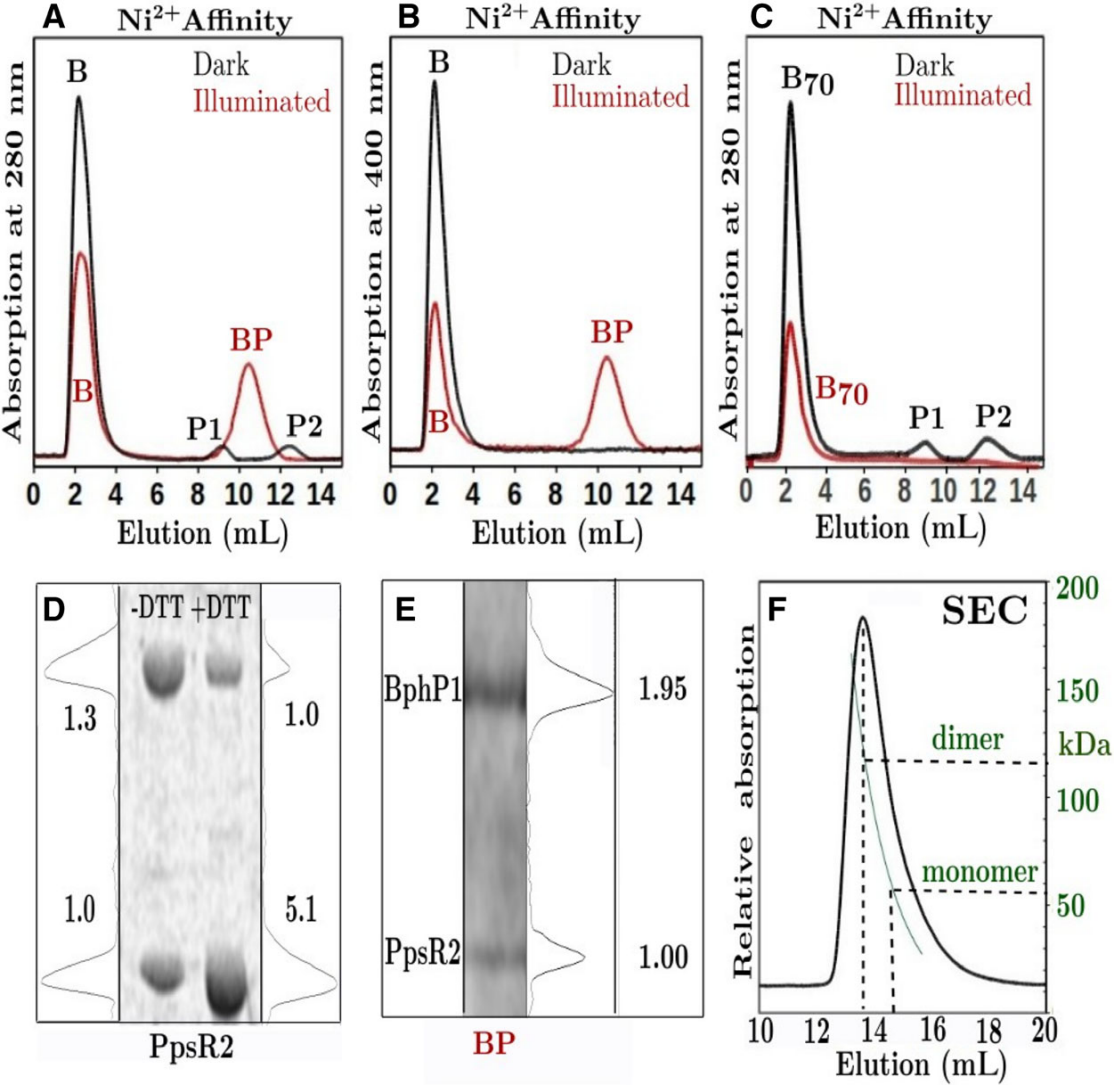
Complex formation by Ni(II)‐affinity chromatography and SDS/PAGE. Ni(II)‐affinity plots (A–C) of samples incubated in the dark (black) or after illumination with light of wavelength 760 nm for 5 min (red). Samples were of untagged *Rp*BphP1 or untagged *Rp*BphP1ΔHOS mixed with N‐terminal His_6_‐tagged *Rp*PpsR2 in the protein ratio 4 : 1. Profiles (A) and (C) were monitored at the protein absorption wavelength 280 nm. (B) was measured at the biliverdin IXα absorption wavelength 400 nm. Protein peak B is full length *Rp*BphP1 and B_70_ the 70 kDa fragment *Rp*BphP1ΔHOS and in the dark both elute at imidazole concentrations of 10 mm, while peak P1 and peak P2 are the monomeric and dimeric His_6_‐tagged *Rp*PpsR2 eluting at 212 and 378 mm imidazole respectively. On illumination with 760 nm light the *Rp*BphP1‐*Rp*PpsR2 complex peak BP is observed eluting at 280 mm, the peak monitored at 400 nm (B) shows that this peak contains *Rp*BphP1. (D) SDS/PAGE gels of *Rp*PpsR2 without (‐DTT) and with (+DTT) 5 mm dithiothreitol. DTT breaks the disulfide bond between HTH domains. Linear scans of ‐DTT gels quantify *Rp*PpsR2 with disulfide bonds and free cysteine in the ratio of ~ 1.3 : 1 and ~ 1 : 5.1 for +DTT gels. (E) An SDS/PAGE gel of peak BP shows the presence two proteins within the complex in the protein ratio ~ 2 : 1, *Rp*BphP1 of molecular weight ~ 80 kDa and *R*pPpsR2 of molecular weight ~ 50 kDa. (F) A SEC plot of *Rp*PpsR2 in 5 mm DTT indicating a dimer of mass ~ 117 kDa and a small tail which may indicate monomers at ~ 50 kDa.

Bacterium *Rps. palustris* photosynthesizes anaerobically and, in strain CGA009, low oxygen conditions have been shown to weaken the binding of photosynthesis repressor proteins PpsR to specific DNA promoter regions. It has also been shown that far‐red light antagonizes binding of *Rp*PpsR2 to DNA promoter regions on photo‐conversion of *Rp*BphP1 to the active Pr state 2, 4. A combination of redox and light control ensures that photosynthetic apparatus is switched on under optimal conditions 4, 30. Under reducing conditions, *Rp*PpsR2 is observed to be predominantly a dimer in solution (Fig. 5F) and not a tetramer, as observed in *Rba. sphaeroides* 22. SDS/PAGE experiments (Fig. 5D) show that, in a 5 mm DTT buffer, ~ 80% of intermolecular disulfide bonds are broken and dimers are free to monomerize. Ni‐affinity chromatography was employed to monitor complex formation between untagged full‐length *Rp*BphP1 or untagged *Rp*BphP1ΔHOS and N‐terminal His_6_‐tagged *Rp*PpsR2 (Fig. 5A–C). Ni‐affinity experiments were performed in the dark and after stimulation with monochromatic 760 nm light. Passing dark adapted mixtures of untagged *Rp*BphP1 (B) and His_6_‐tagged *Rp*PpsR2 (P) through a Ni^2+^ column shows that B does not bind, eluting at buffer imidazole concentration of 10 mM, while P elutes as two peaks, at imidazole concentrations P1 = 210 mm and P2 = 378 mm, corresponding to monomeric and dimeric protein respectively. A 4 : 1 ratio B : P mixture, exposed to 760 nm light, elutes at 10 mm imidazole as a slightly reduced B peak and a BP peak at 280 mm imidazole concentration (Fig. 5A). BP is clearly a complex formed between B and P, as can be seen from eluted fractions monitored with 400 nm light, which is solely a biliverdin absorption wavelength (Fig. 5B). By comparison, *Rp*BphP1ΔHOS (B_70_) cannot form complexes with *Rp*PpsR2, as confirmed by the absence of a 400 nm absorbing band at high imidazole concentrations (Fig. 5C). SDS/PAGE of peak BP shows that it separates into two peaks of molecular weight ~ 80 kDa (*Rp*BphP1) and ~ 50 kDa (*Rp*PpsR2), respectively, as shown in Fig. 5E. A linear SDS/PAGE absorption scan indicates that the *Rp*BphP1:*Rp*PpsR2 complex has a protein ratio of ~ 2 : 1 (Fig. 5E) suggesting that a complex is formed between one *Rp*BphP1 dimer and one *Rp*PpsR2 monomer. A complex of two *Rp*BphP1 dimers and one *Rp*PpsR2 dimer cannot be ruled out. However, BP elutes at imidazole concentration of 280 mm which is closer to monomeric P1 than to dimeric P2, indicating that BP complexes probably bind to a Ni‐affinity column through only one His_6_ tag ligand. Moreover, because 20% of *Rp*PpsR2 disulfide bonds remain oxidized, a 110 kDa, SDS/PAGE peak would be expected if dimers were involved and this is not observed.

## Discussion

### Light‐dependent properties of full‐length bacteriophytochromes

Canonical type 1 BphPs contain two dimerization interfaces, a hydrophobic N‐terminus interface, formed between helices hA, hB, and hE in the chromophore binding domain (CBD) and the C‐terminus Dhp dimerization domain 31. In *Rp*BphP1, as the N‐terminus interface is hydrophilic, only the C‐terminus dimer interface exists. SAXS data suggest that, on photo‐conversion to the Pr state, a structural rearrangement occurs at the C terminus of *Rp*BphP1, which indicates weakening of the dimer interface, but dimers are still observed, and this is likely to be because the short four α–helix bundle remains. Light‐induced structural changes, common to all BphP, are the structural rearrangements within the biliverdin pocket which are transmitted through the PCD domain and the long hI helix. Further structural changes are dependent on the type of BphP and the specific nature of C‐terminus domains. It is therefore of interest to discuss how this differs amongst various full‐length BphPs.

Bacteriophytochrome *Dr*BphP from *Deinicoccs radiodurans* is a type 1 BphP exhibiting a Pr dark state. The photo‐conversion of *Dr*BphP from Pr to Pfr states has been investigated by time resolved SAXS over the range 10 μs to 500 ms 32 and significant differences were observed in Pfr and Pr SAXS envelopes even though radii of gyration values changed by < 0.5 Å. The SAXS envelopes showed a rotation of the catalytic kinase domains about the dimer axis of ~ 50°. An alternative picture has emerged via single particle negative stain electron microscopy 33. Two species of photo‐converted particle were observed, Pfr and Pfr′, which are shortened (143 Å to 124 Å) and broadened by a rotation of PHY domains by 35° away from the two‐fold axis. Other differences were observed at the HK domains, whereas in the Pfr particles, a partial disruption and separation of HK dimers was observed in the Pfr′ particle HK dimers appear to be completely flexible and are practically invisible in EM micrographs. The observed disruption of HK domains suggests a change in the cross‐over angle of hI helices forcing HK domains apart. This is in contrast with what is observed in *Rp*BphP1, where it is the Pr state that is destabilized, but this may only be a reflection of the fact that it is the dark states that are structurally stable in bathy BphPs.

*Is*PadC has a diguanylyl cyclase (DGC) C‐terminus domain that modifies the second messenger molecule c‐di‐GMP. Structural asymmetry is observed in the X‐ray structure of *Is*PadC_reg2_ which has one monomer in the Pr state and the other in the Pfr state. Mutations that stabilize the mixed state were engineered within the 25‐residue coiled‐coil region (Reg2). In the Pfr protomer, Reg2 mutations have the effect of straightening the coiled‐coil, pushing its DGC domain closer to the two‐fold axis. This mutant is catalytically very active, suggesting that asymmetry may be important to its function. Comparing the structures of *Is*PadC and *Rp*BphP1 shows that the Reg2 region maps directly onto the last 25 residues of helix hI in *Rp*BphP1. Bowing of hI helices was observed in *Xcc*BphP1‐Pr suggesting that changes in conformation here is also important in type 3 BphPs. It has been proposed that asymmetry brings DGC monomers closer together, which renders the DGC dimer more active. The movement of Pfr DGC domain toward the two‐fold axis is similar to that observed for *Rp*BphP1 where PAS9 domains are also closer to the two‐fold axis in Pfr than in Pr. It is interesting that *Is*PadC SAXS data of native protein show large changes in the Rg and Dmax, which are 49–56 and 160–170 Å for the dark Pr and the light Pfr states respectively. The SAXS data indicate that, in both *Is*PadC and *Rp*BphP1, Pfr states form more elongated dimers than for Pr. Based on the UV‐Vis absorption spectra, which show incomplete photo‐conversion, a mixed Pr/Pfr state would be expected in the SAXS light‐illuminated data. However, a SAXS envelope has not been shown for this data. Such a calculation, if possible, would provide important information on the degree of asymmetry in the native protein.

Although comparison between different BphP types is useful, there are structural differences within the PCD that complicate matters. For example, the canonical dark‐stable Pr state, in type 1 BphPs, has bowed helices joining PAS‐GAF to PHY domains resulting in a large hole, whereas in other BphP types these helices are relatively straight, causing closer contacts within the dimer spine. It is interesting that the canonical *Dr*BphP has shorter Pfr dimers but Pfr dimers in both *Is*PadC and *Rp*BphP1 are more elongated. This is despite *Is*PadC and *Dr*BphP both being Pr dark stable, whereas *Is*PadC and *Rp*BphP1 have Pr and Pfr dark stable states, respectively, yet exhibit similar SAXS Pfr properties. Structural differences in Pr and Pfr states probably also depend on the very different functional requirements of C terminus domains which are; for type 1 *Dr*BphP a movement of Kinase domains toward the Dhp, in type 5 *Is*PadC it is the bringing together of DGC domains to make catalytic active dimers and in type 3 *Rp*BphP1 a separation of HOS domains to facilitate the formation of complexes with *Rp*PpsR2.

SAXS experiments were designed to obtain pure Pfr and Pr samples and to enable easier data analysis and *ab‐initio* reconstruction of envelopes. However, in a real‐world situation, light will be distributed across the full red to far‐red energy spectrum. There is, therefore, no reason to suppose that mixed states could not exist for *Rp*BphP1. One possible model of the complex will be presented below, which could apply to both pure Pr dimers and mixed Pfr:Pr dimers.

### HOS dimerization interface

HOS domains are essential for the assembly of head‐to‐head parallel dimers through the formation of a C‐terminus four α‐helix bundle that has the same dimer formation function as do Dhp domains in type 1 BphPs. This similarity may point to a common molecular ancestor, for example, type 3 BphP may have evolved from a type 1 BphP by insertion of a PAS9 domain and the loss of a kinase domain with additional point mutations that change Dhp domains to HOS domains. An important difference is that α‐helix hI is continuous with Dhp helices in type 1 BphPs 14 whereas an intervening PAS9 domain disrupts this helix in *Rp*BphP1. Although precise details on HOS contacts cannot be determined using the SAXS data, modeling nevertheless suggests that hJ′ helices must overlap with hI helices so that the long four α‐helix bundle can fall within the Pfr envelope. An interesting feature of our Pfr model is that helix hJ is brought in line with helix hI, creating a spatial helical topology that is similar to type 1 BphP, albeit with a break between these helices caused by an intervening PAS9 domain. The Pr model best fits the SAXS data when helices hJ and hK occupy the bulge region between PHY and PAS9 domains and this can be achieved through the hinge region which allows helices hJ and hK to articulate independently while leaving helices hJ′ in situ to form a short four helix bundle with hI helices. The Pfr model is consistent with the observation that the Pfr state is functionally inert, and we propose that this is because the long four α‐helix bundle confers structural stability to the HOS dimer while making HTH motifs unavailable for complex formation with *Rp*PpsR2. Alternatively, our Pr model has free hJ and hK helices that can interact with *Rp*PpsR2. The corollary of this is that an HOS deleted construct is incapable of forming complexes with *Rp*PpsR2.

### A mechanism for HOS domain activation

**Figure 6:**
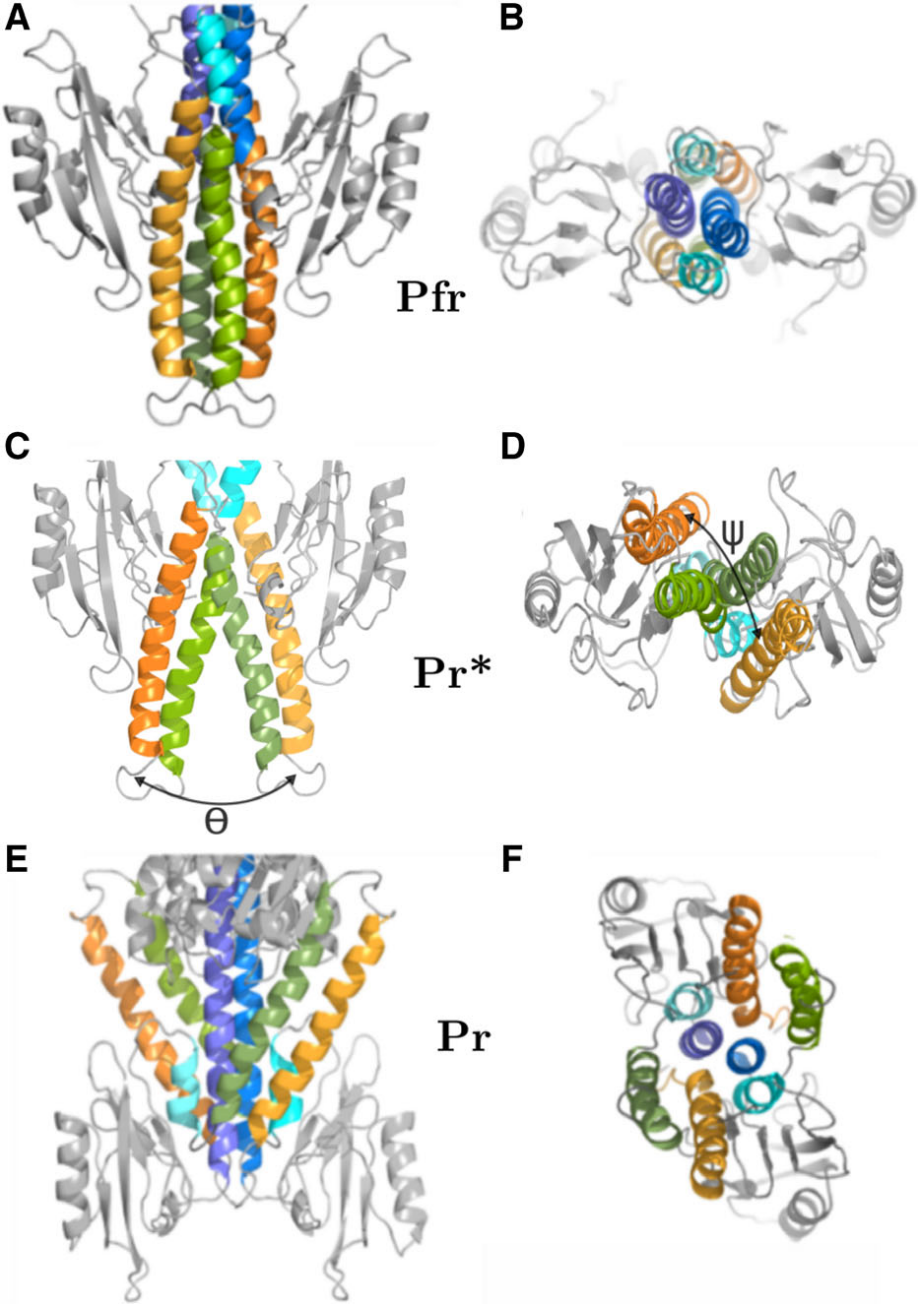
HOS domain transition from Pfr to Pr state. The proposed HOS domain transition from Pfr (A and B) to Pr (E and F) via an intermediate Pr* (C and D) model (front and top views). Pfr‐HOS dimers form a four α‐helix bundle (hJ‐hK)_A_‐(hJ‐hK)_B_ where A and B are the dimer protein chains. The intermediate model Pr* shows the beginning of α‐helix bundle separation and was generated from transformation matrices calculate by superimposition of PAS9 of Pfr onto Pr and applied to each Pfr PAS9‐HTH structure in turn. Transition from Pfr to Pr* can be described as a two angle motion of HTH motifs (hJ‐hK)_A_ and (hJ‐hK)_B_; the first is a separation angle θ ~ 20° and the second a shearing angle ψ ~ 15°. We propose that this is facilitated by bending at the hinge. PAS9 domains (gray), helix hI (blue), short α‐helix hJ′(cyan), hJ (green) and hK (orange). The two subunits A and B are colored in darker and lighter shades respectively.

There are a number of studies characterizing photo‐induced structural changes in the PCD domain which can be used to propose a tentative mechanism for HOS domain activation in the Pr state. Light excitation of biliverdin IXα causes isomerization of the D pyrrole ring and rearrangement of amino acid residues in the biliverdin pocket 8, 34. These changes are transmitted to a tongue motif (466–476) capping the biliverdin pocket where an α‐helix unfolds into a β‐strand on photo‐conversion from the Pfr to Pr state. A two‐stranded β‐sheet is formed with β‐strand 439–448, which elongates the tongue motif, pushing the PHY domain toward the C terminus 11, 35. These first light‐induced structural changes characterize the transition from Pfr to Pr states and appear to be common to all BphPs. In type 3 *Xcc*BphP‐Pr, the movement of PHY domain toward the C terminus causes curvature of α‐helix hI and a rotation of the PAS9 domain away from the twofold axis 15. A consequence of this motion could be the weakening of interactions between HTH motifs. Hence, we propose that they separate and move independently toward their respective PHY domains through a rearrangement of conformation in the hinge region. Although SAXS data do not permit precise modeling of four α‐helix bundle contacts, large movements of HTH motifs can be observed in the data which suggests that they must separate. Figure 6 provides a description of a possible mechanism for the transition from Pfr to Pr states. Whereas in our model a short and long four α‐helix bundle stabilizes the Pfr dimer, making it functionally inert (Fig. 6A,B), the Pr dimer is only stabilized by a shorter four α‐helix bundle (hJ′‐hI)_2_, as shown in Fig. 6E,F. To illustrate how the rotation of PAS9 may cause HTH separation, an intermediate conformation, Pr*, is shown assuming that HTH domains follow the movement of PAS9 domains. The intermediate structure can be calculated from transformation matrices defining PAS9 movement from Pfr to Pr positions and applying these to their respective Pfr HTH motifs (Fig. 6C,D). The motions of HTH motifs can be described as a radial angular separation of θ ~ 20° and a shearing motion of ψ ~ 15°, resulting in a separation between HTH motif turns (T) of ~ 20 Å. The formation of four α‐helix bundles is energetically favorable due to hydrophobic and helix dipole interactions. However, if HTH motifs are forced apart, other factors can dominate, such as the minimization of amino acid conformation energies within the hinge region, which in the absence of any other constraints can result in large movements of HTH motifs. A feature of the Pr model is that HTH motifs are released from association within a four α‐helix bundle so that their sticky inner helix surfaces are exposed and able to form a complex with *Rp*PpsR2.

### An *Rp*BphP1‐*Rp*PpsR2 complex model

**Figure 7:**
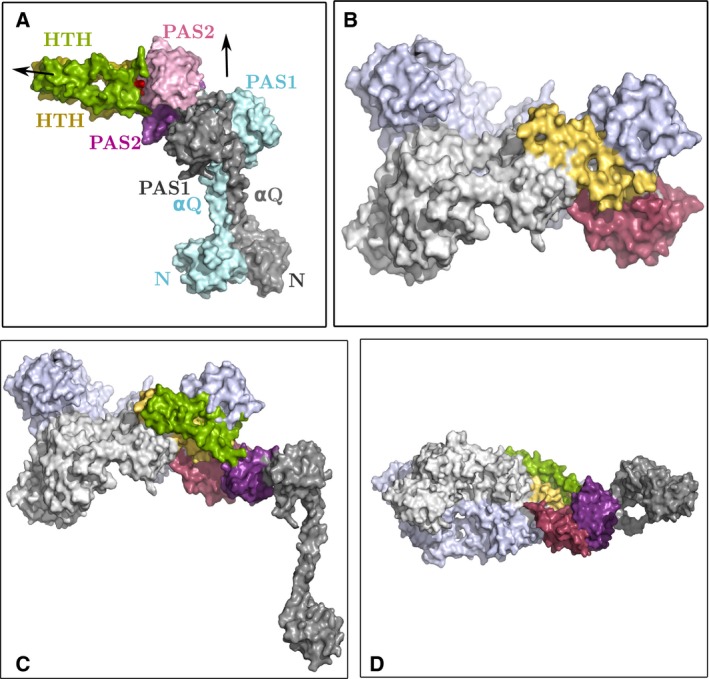
Space filling model showing a possible (*Rp*BphP1)_2_ *Rp*PpsR2 complex. (A) space filling model of the full length *Rp*PpsR2 dimer synthesized in Modeller using a sequence modified PpsR homolog structure, 4HH2, from *Rba. sphaeroides* missing the C terminus HTH domain. The missing HTH domain was built in Modeller from PAS‐HTH and HTH homolog structures, 4GCZ, 3A0R, 4I5S, 1NTC and 3E7L. The PAS domain of PAS‐HTH overlaps with the PAS2 domain of 4HH2 facilitating alignment of the full‐length structure. Arrows in (A) indicate two‐fold axes relating N‐αQ‐PAS1 and PAS2‐HTH respectively the latter is approximately at right angles to the first. One monomer is colored (gray, purple, green) the other (cyan, pink, yellow). HTH domain disulfide bond is colored red. (B) Space filling model of *Rp*BphP1‐Pr dimer, PAS9 (red) and HOS (yellow) are part of one monomer the rest of *Rp*BphP1 is colored pale gray or pale blue for each subunit. (C) front view of hetero‐complex (*Rp*BphP1)_2_‐*Rp*PpsR2 in which contacts are made between HTH motif of HOS (yellow), HTH of an *Rp*PpsR2 monomer (green), contacts are also made between PAS9 (red) and PAS2 (purple) domains. (D) is as in (C) but viewed from the bottom.

SAXS and biochemical data suggest one possible *Rp*BphP1:*Rp*PpsR2 complex model that is consistent with experimental results, although the model presented here should be treated with caution until hard structural data become available. Nevertheless, it is worthwhile discussing the constraints that observed data can impose on complex models. These constraints are: (a) the formation of a complex with a protein ratio *Rp*BphP1:*Rp*PpsR2 of 2:1, (b) a central role played by HOS domains in the formation of complexes which also explains why *Rp*BphP1ΔHOS cannot make complexes, and (c) complexes must be able to form with the Pr and not with the Pfr form of *Rp*BphP1. A model was built based on these constraints and from the full‐length structures of *Rp*BphP1 and *Rp*PpsR2. A full‐length model of *Rp*PpsR2 was based on a homolog partial structure *Rs*PpsRΔHTH (PDBID 4HH2) from *Rba. sphaeroides* which represents ~ 83% of the full‐length molecule and has ~ 28% sequence identity with *Rp*PpsR2. The missing part of the structure is the HTH domain at the C terminus belonging to the FIS family of DNA‐binding proteins which form four α‐helix bundles. A number of homologous HTH domains were found using the online toolkit *HHpred* and these were used to build *Rp*PpsR2 in Modeller (Fig. 7A). Structures were of the HTH or PAS‐HTH type, and the latter proved useful for alignment because the PAS domains overlap with the PAS2 domain of *Rs*PpsRΔHTH. The *Rp*PpsR2 model has two distinct twofold axes, the first relating N‐αQ‐PAS1 domains and the second approximately perpendicular to the first relating PAS2‐HTH domains.

A model of the complex that satisfies the aforementioned constraints has an *Rp*BphP1‐Pr dimer forming a complex with a single *Rp*PpsR2 monomer. Combined constraints of a 2 : 1 complex protein ratio and HOS domains that play a direct role in complex formation require that HTH motifs form hetero‐dimer four α‐helix bundles such that one HTH is donated from an *Rp*BphP1 dimer (HTH_B_) and the other from an *Rp*PpsR2 monomer (HTH_P_). Two forces are involved in the formation of four α‐helix bundles, electrostatic anti‐parallel helix dipoles and hydrophobic forces between inner α‐helix surfaces. These inner surfaces are usually lined with small hydrophobic residues, such as Leu, Ala, Ile, and Val. However, homo dimers must separate for these hydrophobic surfaces to be available for interaction and so that hetero dimers are formed. This is the case in the *Rp*BphP1‐Pr model as HTH motifs are bent back whereby hydrophobic surfaces point outwards (Fig. 7B), as well as for *Rp*PpsR2, which can be monomerized when the inter‐molecular disulfide bond is broken. A model of the complex is shown in Fig. 7C,D. An unexpected feature of the model is that the PAS9 domain of *Rp*BphP1 and the PAS2 domain of *Rp*PpsR2 naturally come into contact with one another. Proteins with dimeric PAS‐HTH domain architecture are interesting because they are known to form both homo and hetero dimers. In these structures, HTH domains serve as dimerization domains, while PAS domains provide stability and specificity to the dimer 36. Heterodimers are well‐documented, such as the ARNT protein heterodimer with AHR 37 and mammalian hypoxia‐inducible factor 38 or dioxin receptor, which makes homo dimers as well as heterodimers with ARNT 39. A feature of the proposed complex model is that it can work with pure Pr dimers and mixed Pfr/Pr *Rp*BphP1 dimers.

*Rps. palustris* CGA009 is a model strain that has been extensively used for genomic sequencing and other biochemical experiments. Unfortunately, in this strain, gene *bphP1* is frame shifted and not expressed. Thus, for this work, *bphP1* was PCR amplified from strain 2.1.6 which has an identical gene sequence as that of *bphP1* in strain CGA009 if the frame shift is repaired 40. In fact it has been shown that a repaired *bphP1* gene is functionally a far‐red light sensor and that it targets the repressor protein *Rp*PpsR2 in a redox sensing way 4. However, strain CGA009 *Rp*PpsR2 differs from all other DNA sequenced wild type strains (> 10) which do not have a redox Cys in the HTH domain and are thus non‐redox sensing forms of PpsR. Consequently, in most strains, RpPpsR2 is normally only under the control of light by interaction with *Rp*BphP1. It has been suggested that CGA009 is a laboratory strain which has acquired a frame‐shifted *bphP1* and an R428C *Rp*PpsR2 mutation over time due to laboratory selection of pigmented bacteria under certain laboratory conditions, which highlights the pitfalls of using laboratory strains 41. The work presented here on complex formation between a spectrally active *Rp*BphP1 and an *Rp*PpsR2 in reducing conditions is therefore a good model of complex formation in wild‐type strains. Although wild strains of *Rp*PpsR2 are not redox sensors, in *Rps. palustris*, photosynthesis remains under redox control through other means. For example, the cytochrome c2 encoding gene *cycA* transfers electrons to the Reaction Centre from outside the cell and the redox controlled transcription regulator encoding gene *aerR* has promoter regions which are recognized by PpsR2. Also, PpsR2 controls the expression of PpsR1 which is a redox sensor and binds to a number of promoter regions within the major cluster of photosynthetic genes. There is therefore a complex interplay between light‐dependent antagonistic action of *Rp*BphP1 on *Rp*PpsR2 and redox potential on *Rp*PpsR1 but not on *Rp*PpsR2 19. A fine modulation and control of photosynthesis has therefore been achieved in this bacterium by partitioning light‐ and redox‐dependent gene expression between two different PpsR molecules.

## Materials and methods

### Cloning and purification

*G*ene *rpbphP1* was designed *in silico* to be used in spectroscopy, size exclusion chromatography and Ni^2+^ affinity column binding experiments. The gene was synthesized by GenScript (Piscataway, NJ, USA) in plasmid pUC57 with *NdeI* restriction site at the 5′ end and sites *HindIII* and *XhoI* at the 3′. The gene was sub‐cloned, using STRU‐cloning protocol 42, into pET28a (*NdeI* and *HindIII*), pET24a (*NdeI* and *HindIII*) and pET24a (*NdeI* and *XhoI*) to obtain expression constructs for N‐terminal His‐tag, C‐terminal His‐tag and untagged *Rp*BphP1 protein respectively. For SAXS experiments gene *rpbphP1* was PCR amplified from genomic DNA of bacterium *Rhodopseudomonas palustris strain* 2.1.6 and sub‐cloned into pET28a. Transformed *E. coli* BL21 (DE3) cells were grown to a density of OD_600_ = 0.6, induced with 0.02 mm IPTG and incubated overnight at 18 °C. Cells were harvested and re‐suspended in chromatography buffers containing 10 mm Biliverdin IXα (Sigmal‐Aldrich Company Ltd, Dorset, UK), protease inhibitor tablets (Roche Diagnostics GmbH, Mannheim, Germany), DNaseI (10 μg·mL^−1^), Triton X‐100 (0.5%) and disrupted by French Press. Supernatants were loaded onto an affinity His‐Trap™ HP or ion exchange HiTrap QFF columns (GE Healthcare Bio‐Sciences, Pittsburgh, PA, USA). Finally, proteins were gel filtered using a HiLoad 26/60 Superdex 200 column (GE Healthcare) in a 5 mm TrisHCl pH 8 and 10 mm NaCl buffer. Plasmid pET28a with incorporated gene *ppsR2* from *Rhodospeudomonas palustr*is was kindly provided by Shinji Masuda (Tokyo Institute of Technology) and Tom Beatty (University of British Columbia). Protein was expressed in *E. coli* BL21 (DE3), using the same protocol as *Rp*BphP1, and purified on a His‐Trap HP column followed by gel filtration and stored in a final buffer of 20 mm Tris‐HCl pH 8, 300 mm NaCl, and 5 mm dithiothreitol.

### UV‐visible spectroscopy

UV‐Visible spectra were recorded at room temperature in a Perkin Elmer Lambda 35 UV/VIS spectrometer. Spectra were recorded in the dark or after illumination with monochromatic light produced by passing white light through an interference filter with a bandwidth of 10 nm centered on wavelength 760 nm (Knight Optical Ltd, Harrietsham, Kent, UK). The fiber optic/filter system was placed ~ 4 cm from the sample which provided a photon flux density of ~ 250 μmol·m^−2^·s^−1^ at the sample. There was sufficient light to photo‐convert a Pfr dark state into a 100% Pr state within 1 min. The monochromatic light was passed through the top of the UV‐Vis cuvette, with a sample path‐length of 2 mm, and so full photo‐conversion to the Pr state was achieved over a wide range of protein concentrations, 0.04–4 mg·mL^−1^.

### Gel filtration analysis

Purified protein was loaded onto a Superose 12 10/300 GL in 200 μL volumes at concentrations between 1 and 4 mg·mL^−1^ in 5 mm TrisHCl pH 8 and 10 mm NaCl. For *Rp*BphP1 this was either in the dark or after illumination with 760 nm light for 5 min. The column was calibrated with high molecular weight calibration kit (GE Healthcare). Data were plotted on a logarithmic scale using K_av_, to obtain estimates of protein molecular weight in solution.

### *Rp*BphP1‐*Rp*PpsR2 complex formation analysed by Ni(II) affinity chromatography

N‐terminal His_6_‐tagged *Rp*PpsR2 and untagged *Rp*BphP1 or untagged *Rp*BphP1ΔHOS was mixed in a ratio 1 : 4 respectively. Samples were pre‐incubated in the dark or illuminated for 5 min with light of wavelength 760 nm to achieve a full Pr state. Chromatography was carried out over 10‐20 min using a 1 mL His‐Trap HP column and an ÄKTA Explorer. The elution was monitored at the protein absorption wavelength 280 nm and the biliverdin IXα absorption a wavelength of 400 nm. *Rp*BphP1 can only be eluted at high imidazole concentrations when in complex with His_6_‐*Rp*PpsR2 and monitoring absorption at these two wavelengths allows peaks to be differentiated between those that contain His_6_‐*Rp*PpsR2 only and those also in complex with *Rp*BphP1.

### SAXS data measurement

Small‐angle X‐ray scattering (SAXS) data were collected at STFC SRS facility beam line 2.1 at the wavelength λ = 1.54 Å. A SAXS X‐ray camera was assembled at two different lengths, 1.00 and 4.25 m, to cover scattering ranges 0.052 < S < 0.730 Å^−1^ and 0.014 < S < 0.183 Å^−1^ respectively (Table 1). Samples were washed several times with 5 mm Tris buffer (pH 8) in a Vivaspin 500 centrifugal filter unit (Satorius AG, Goettingen, Germany) to remove chemicals such as salt and imidazole. The sample was concentrated to ~ 4 mg·ml^−1^ and spin column flow through buffer was retained for SAXS data background subtraction. For the longer camera length, the sample was further diluted to 1 mg·ml^−1^. Samples were injected into a 50 μL volume brass cell with X‐ray path length of 1 mm covered on both sides by thin mica windows and chilled to 8 °C by water‐circulation around the brass cell. Data were collected as 60, 1‐min exposure, images on a time resolved multi‐wire area detector, assessed for time dependant radiation damage and averaged. A Pfr dark state was prepared by protein purification in the dark and pre‐incubation in the dark overnight. Pr state sample was induced by continuous illumination with white light passing through a 10 nm bandwidth interference filter centered on 760 nm (Knight Optical (UK) Ltd). The radius of gyration (Rg) was evaluated using the Guinier approximation *I(S) = I(0)exp(−S* ^*2*^ *Rg* ^*2*^ */3)* for *S*.^.^ *Rg* < 1.3 and was also calculated with Dmax by the indirect Fourier‐transform program gnom. Molecular weights were determined with the program saxs mow2 29. For accurate molecular weight the method assumes a mono‐dispersed sample and is also a check on the purity of the sample. The monochromatic light source system, protein concentration, sample path length, and photon flux density used on the SAXS beam line was the same as used in UV‐Vis spectroscopy experiments so that a full photo‐conversion to the Pr state was guaranteed.

### SAXS data processing and modeling

The EMBL program suite atsas (http://www.embl-hamburg.de/biosaxs/software.html) was used for data analysis. *Ab‐initio* envelopes of dark Pfr and illuminated Pr states were calculated from SAXS data in 10 runs, for each state, with the program dammin 43. The programs supcomb, damfilt and damaver were used to calculate average envelopes 44 which were isotropically reduced so that maximum envelope dimensions were the same as Dmax derived from pair distribution functions P(R). Models were initially fitted into SAXS envelopes using the graphics program pymol equipped with the plugin CRYSOL to access the quality of model fit to SAXS data 45. Domain orientations were fine‐tuned using χ^2^ as a measure of fit between calculated and measured SAXS scattering curves. A rigid body refinement program sasref was also used to perform an automated 3D search of domain alignments 46. Discrepancies between experimental and model scattering data were assessed with reduced residuals (*I*(S)_exp_‐*k*.*I*(S)_cal_)/σ(S), as a function of S, where *I*(S)_exp_ are measured intensities and *I*(S)_cal_ are intensities calculated from atomic models, *k* a scale factor and σ(S) are SAXS data standard deviations 47. A second target function, |ΔRg|/σ, was also used which calculates the consistency between calculated and experimental radii of gyration (Rg), and is defined as the modulus difference between Rg values divided by the experimental standard deviation σ and should be less than 1.

### Domain modeling

Atomic models of *Rp*BphP1 HOS and *Rp*PpsR2 HTH domains were built so that full length models of *Rp*BphP1 and *Rp*PpsR2 could be created. Homolog proteins were found with the toolkit *HHPred* 48 (https://toolkit.tuebingen.mpg.de) and sequence aligned to their X‐ray structure sequences. Best homolog structures based on hit probabilities, usually 4 to 6 structures, were selected as PDB templates to automatically build 3D structures with the program modeller 49.

## Conflict of interest

The authors declare no conflict of interest.

## Author contributions

MP‐ conception and design of experiments, writing and critical revision of the manuscript, SAXS data collection, *ab‐initio* envelope determination, modeling of SAXS data and measurement of UV‐Vis spectra. DB‐ cloning and sample purification for SEC measurements, Ni(II)‐affinity chromatography measurement of complex formation, critical reading of manuscript and measurement of UV‐Vis spectra. KE‐ PCR cloning of SAXS *Rp*BphP1 sample, protein purification and characterization of purity by biochemical methods and UV‐Vis spectroscopy. GG‐ collection of SAXS dark and light induced state data, processing of raw images, reduction and analysis of SAXS data and revision of the manuscript. AF‐S‐ assessing quality of Pfr and Pr samples for SAXS experiments, checking SAXS sample purity by SDS/PAGE electrophoresis, UV‐Vis spectroscopy and assisting in SAXS data collection.
